# Antioxidant effects of *Etlingera elatior *flower extract against lead acetate - induced perturbations in free radical scavenging enzymes and lipid peroxidation in rats

**DOI:** 10.1186/1756-0500-4-67

**Published:** 2011-03-17

**Authors:** Tan Jackie, Nagaraja Haleagrahara, Srikumar Chakravarthi

**Affiliations:** 1 Human Biology Division, School of Medicine, International Medical University, Kuala Lumpur, Malaysia; 2 Pathology Division, School of Medicine, International Medical University, Kuala Lumpur, Malaysia

## Abstract

**Background:**

*Etlingera elatior or 'pink torch ginger*' (Zingiberaceae) are widely cultivated in tropical countries and used as spices and food flavoring. The purpose of this study was to evaluate the antioxidant effects of *Etlingera elatior *against lead - induced changes in serum free radical scavenging enzymes and lipid hydroperoxides in rats.

**Findings:**

Rats were exposed to lead acetate in drinking water (500 ppm) for 14 days alone or plus the ethanol extract of *E. elatior *(50, 100 and 200 mg/kg). Blood lead levels, lipid hydroperoxides, protein carbonyl contents and oxidative marker enzymes were estimated. Lead acetate in drinking water elicited a significant increase in lipid hydroperoxides (LPO) and protein-carbonyl-contents (PCC). There was a significant decrease in total antioxidants, superoxide dismutase, glutathione peroxidase and glutathione S-transferase levels with lead acetate treatment. Supplementation of *E. elatior *was associated with reduced serum LPO and PCC and a significant increase in total antioxidants and antioxidant enzyme levels.

**Conclusions:**

The results suggest that flower extract of *Etlingera elatior *has powerful antioxidant effect against lead - induced oxidative stress and the extract may be useful therapeutic agent against lead toxicity. However, detailed evaluations are required to identify the active antioxidant compounds from this plant extract.

## Background

Lead (Pb) is a toxic metal that induces a wide range of behavioral, biochemical and physiological effects in humans. Even though blood lead levels continue to decline over the past two decades, specific populations like infants, young children and working class are still at a higher risk [1,2]. As lead exposure tends to be sub acute, produces only subtle clinical symptoms. Chronic exposure cases are more common than acute toxicity. Lead via gastro intestinal absorption is first taken up by the red blood cells and is distributed to all vascular organs [3]. Pathogenesis of lead poisoning is mainly attributed to lead- induced oxidative stress. Chronic lead exposure is known to disrupt the pro oxidant/antioxidant balance existing within the mammalian cells [4,5]. Lead is reported to cause oxidative stress by generating the release of reactive oxygen species (ROS) such as superoxide radicals, hydrogen peroxide and hydroxyl radicals and lipid peroxides [6-8].

As oxidative stress has been mainly implicated in the lead toxicity, reducing the possibility of lead acetate interacting with cellular metabolism biomolecules and decreasing the reactive oxygen species generation by the use of antioxidant nutrients has received considerable attention in the recent past [4,9,10]. There has been increased interest among phytotherapy researchers to use medicinal plants with antioxidant activity for protection against heavy metal toxicity [6,8].

*Etlingera elatior*, also known as 'torch ginger' or 'red ginger lily' belongs to zingiberaceae family and is a herbaceous perennial plant native to South East Asia. It is known as *bunga kecombrang *or *honje *in Indonesia and as *bunga kantan *in Malaysia. Flowers of *E. elatior *are in shades of pink and red and the inflorescence of the plant are borne on erect stalk of the plants. More than 15 species of *Etlingera *plants have been recorded in Peninsular Malaysia [11,12]. The young shoots and flower buds of the plants are consumed raw by indigenous communities in Malaysia and Thailand. Inflorescence of *E. elatior *is used for flavoring the food and also as ornamentals. The flowers and flower buds are commonly used in Malaysian dishes such as, *Penang laksa*, *nasi kerabu *and *nasi ulam *[11,13-15].

*E. elatior *has been well known for its medicinal properties among indigenous communities in Malaysia. Decoction prepared from the fruit of *E. elatior *has been used to treat ear ache and the leaves have been used in wound healing. The young flower shoot of *E. elatior *was reported to have antimicrobial, cytotoxic and anti-tumor promoting properties [16]. *E.elatior *inflorescence is known to have high antioxidant properties [17,18]. Literature is scanty regarding the phytochemical studies conducted on the inflorescence of *E. elatior*. Most of the previous studies on the antioxidant activities of *E. elatior *were limited to rhizomes and leaves. There are no reports available on the antioxidant activities of inflorescence of *E. elatior *against lead induced toxicity. Hence the present study was taken up to assess the effects of ethanol extract of *E. elatior *flower on serum free radical scavenging enzymes and lipid hydroperoxides in lead acetate - induced toxicity in rats.

## Methods

### Chemicals and Plant material

Lipid hydroperoxide (LPO), protein carbonyl content (PCC), superoxide dismutase (SOD), glutathione peroxidase (GPX), glutathione S-transferase (GST) assay kits were purchased from Cayman Chemicals (Cayman Chemicals and Pierce Biotechnology, USA). Lead acetate (99.5%) was purchased from Sigma Chemical Co. (St. Louis, MO, USA). The inflorescence of the *Etlingera elatior *plant was used in this research because it is eaten by the local population. Twelve kg of *Etlingera elatior *inflorescence was collected from a nursery in Sungai Buloh, Kuala Lumpur, Malaysia, in August 2008 and was authenticated by Department of Horticulture, University Putra Malaysia. The voucher specimen was deposited in the research laboratory at International Medical University (voucher specimen: IMU/PMR/2008/01).

### Plant extraction

Inflorescence of *Etlingera elatior *washed in running tap water three times and cut into 3 cm pieces and again washed and soaked in running tap water for five minutes then air dried. The fresh, dried petals (500 g) were extracted thrice with 95% ethanol (1:10 w/v) using a soxhlet apparatus set at 50°C for 3 hrs. The extract was filtered, evaporated in vacuum evaporator and lypholized to give yield of about 60 g of dry extract. The final product was a pink/purplish fine powder that bears the ginger aroma. The powder was stored in 50 ml polypropylene tubes away from direct light sources at 4°C till further use. The extract was reconstituted with distilled water to give desired concentrations used in this study.

### Phytochemical evaluation

The *Etlingera elatior *inflorescence extract was tested for the presence of various phytochemical classes of compounds such as alkaloids, phenolic compounds, flavonoids, tannins, and saponins using standard procedures of analysis [19,20].

#### Test for alkaloids

1 cm^3 ^of HCl was added to 3 cm^3 ^of each extract in a test tube. The mixture was heated for 20 minutes, cooled and filtered. 2 drops of Wagner's reagent was added to 1 cm^3 ^of the filtrate and observed for reddish brown precipitate.

#### Test for tannins

(a) 1 cm^3 ^of freshly prepared 10% KOH was added to 1 cm^3 ^of each of the extracts and observed for dirty white precipitate.

(b) Two drops of 5% FeCl_3 _was added to 1 cm^3 ^of the extracts and observed for green precipitate.

#### Test for flavonoids

To 3 cm^3 ^of each extract was added 1 cm^3 ^NaOH and observed for yellow colouration.

#### Test for glycosides

10 cm^3 ^of 50% H_2_SO_4 _was added to 1 cm^3 ^of the extract in a test tube. The mixture was heated in a boiling water-bath for 15 minutes. 10 cm^3 ^of Fehling's solution was added and the mixture was boiled and observed for brick red precipitate.

#### Test for saponins

2 cm^3 ^of each extract in a test tube was vigorously shaken for two minutes and observed for persistent foaming.

### Animals and Experimental design

Three months old male Sprague Dawley rats weighing 180 - 200 g were purchased from University Kebangsaan Malaysia (UKM), Kuala Lumpur, Malaysia, and housed under standard laboratory conditions (25 ± 2°C; 12 h light/dark cycles). The rats had access to an animal diet and tap water *ad libitum*. The rats were placed in polypropylene cages with three animals per cage and were allowed to acclimatize for one week prior to treatment.

All the experimental protocols conducted on rats were performed in accordance with the internationally accepted principles for laboratory animal use and care and Institutional animal care and use committee and the study got approval from the Research and Ethics committee.

Animals were divided randomly into the following groups with eight animals in each group. Group 1: control; Group 2: rats exposed to lead acetate in drinking water (500 ppm); Groups 3-5: received E. elatior (50, 100 and 200 mg/kg b.w). Group 4: Rats exposed to lead acetate and treated with *E. elatior*. Groups 6 - 8: received lead acetate in drinking water and *E. elatior *at the same dose daily for 14 days. *E. elatior *extract was diluted with distilled water to the desired concentration of 50, 100 and 200 mg/kg body weight and was orally administrated to rats (0.5 ml/rat/day).

At the end of experimental period, rats were anaesthetized with diethyl ether and blood was collected by cardiac puncture. Serum was separated and used for the determination of lipid hydroperoxides (LPO), total antioxidants, protein carbonyl content (PCC), superoxide dismutase (SOD), glutathione peroxidase (GPx) and glutathione S- transferase (GST). All biochemical parameters were assayed according to the standard procedures using ELISA kits (Cayman Chemicals and Pierce Biotechnology USA). Whereas, protein levels were estimated by protein assay kits from Cayman Chemicals (Cayman Chemicals and Pierce Biotechnology, USA). Furthermore, blood lead levels were assayed by graphite furnace atomic absorption spectrophotometry method.

#### Total antioxidant assay

Using the total antioxidant assay kit, aqueous and lipid soluble antioxidants were not separated and thus combined antioxidant activities of all its constituents were assessed. The assay relies on the ability of antioxidants in the sample to inhibit the oxidation of ABTS (2, 2'-Azino-di-[3-ethylbenzthiazoline sulphonate]) to ABTS^®·+ ^by metmyoglobin. The amount of ABTS produced was monitored by reading the absorbance at 405 nm.

#### Lipid Hydroperoxide (LPO) assay

A quantitative extraction method as provided in the kit method for lipid hydroperoxide assay was used to extract lipid hydroperoxides into chloroform and the extract was directly used. This procedure eliminates any interference caused by hydrogen peroxide or endogenous ferric ions in the sample and provides a sensitive and reliable assay for lipid peroxidation. The absorbance was read at 500 nm using a 96 well plate spectrophotometric reader and a dose response curve of the absorbance unit vs. concentration in nmol was generated.

#### Protein carbonyl content (PCC) assay

In the protein carbonyl content assay kit, protein samples are derivatized by making use of the reaction between 2, 4-dinitrophenylhydrazine (DNPH) and protein carbonyls. Formation of a Schiff base produces the corresponding hydrazone which was analyzed spectrophotometrically at 360 - 385 nm.

#### Superoxide dismutase (SOD) assay

This assay kit utilizes a tetrazolium salt for the detection of superoxide radicals (O_2_^-^) generated by xanthine oxidase and hypoxanthine. One unit of SOD is defined as the amount of enzyme necessary to exhibit 50% dismutation of the superoxide radical. Oxidation rate of tetrazolium salt to formazan dye by O_2_^- ^is inversely proportional to the endogenous activity of SOD. The formazan dye stains the wells and its staining intensity was detected by absorbance spectrophotometry at 450 nm using a plate reader.

#### Glutathione peroxidase (GPx) assay

This assay kit measured GPx activity indirectly by a coupled reaction with glutathione reductase. Oxidized glutathione, produced upon reduction of an organic hydroperoxide by GPx, is recycled to its reduced state by glutathione reductase and NADPH. The oxidation of NADPH to NADP^+ ^is accompanied by a decrease in absorbance at 340 nm. The rate of decrease in the absorbance at 340 was directly proportional to the GPx activity in the sample.

#### Glutathione S-transferase (GST) assay

The Cayman Chemical GST assay kit measured total GST activity (cytosolic and microsomal) by measuring the conjugation of 1-chloro-2, 4-dinitrobenzene (CDNB) with reduced glutathione. The conjugation is accompanied by an increase in absorbance at 340 nm. The rate of increase is directly proportional to the GST activity in the sample.

### Data analysis and statistics

Data were expressed as mean ± standard deviation. Statistical analysis was done by Graph Pad Prism 5.0 software package. Statistical differences among various groups and the significance were calculated by non-parametric Kruskal Wallis and Mann- Whitney's U-Tests. P value less than 0.05 was considered statistically significant.

## Results

### Phytochemical screening

Phytochemical screening study of *Etlingera elatior *inflorescence extract revealed that the extract had significant quantity of phenolic compounds and flavonoids but no tannins, alkaloids and saponins were detected (table 1). The total phenolic content of the ethanolic extract was 424 ± 62 mg gallic acid equivalent (GAE) per gram of extract. The total flavonoid content of the extract was 772 ± 34 mg GAE per g of extract.

**Table 1:** The analysis of phytochemicals in the ethanolic extract of inflorescence of *E. elatior*

Phytochemicals	Ethanol Extract of *E. elatior*
Alkaloids	-

Glycosides	+

Tannins	-

Saponin	-

Flavonoids	++

Phenols	++

+ Presence of compound in moderate amount; ++ Presence of compound in significant amount; - absent.

### Blood lead levels

**Figure 1:**
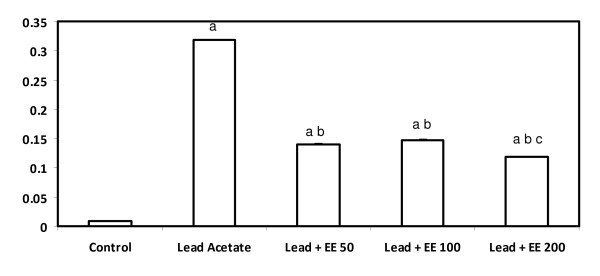
**Blood lead levels (μg/L) in rats in different experimental groups**. Results are expressed as means ± S.D. of eight rats per group; EE - *Etlingera elatior *a - Significantly different from control - p < 0.05 b - Significantly different from lead alone treatment group - p < 0.05 c - Significantly different from lead + EE group - p < 0.05

There was a significant increase in lead level in the serum of rats in the lead acetate alone-treated group. Whereas, no significant differences were found between the groups, that received the combined treatment or lead acetate and the extract at 50 and 100 mg. Moreover, animals treated with lead acetate plus the extract at the highest dose (200 mg) showed a significant decrease in serum lead level compared to the other treated groups (Figure 1).

### Total antioxidants

**Figure 2:**
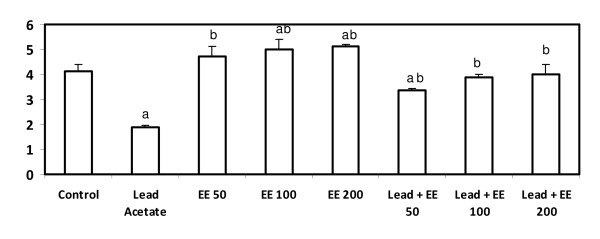
**Serum total antioxidants level (μmol/mg protein) in control and *E. elatior *alone or in combination with lead acetate-treated rats**. Results are expressed as means ± S.D. of eight rats per group; EE - *Etlingera elatior *a - Significantly different from control - p < 0.05 b - Significantly different from lead alone treatment group - p < 0.05

Animals treated with lead acetate alone showed a significant decrease in serum total antioxidants (p < 0.05) whereas, those treated with the extract alone at the three tested doses showed a significant increase in total antioxidants. Animals received the combined treatment of lead acetate and the extract at the three tested doses showed a significant improvement in total antioxidants (p < 0.05). This improvement was more pronounced in the group received lead acetate and treated with the highest dose of *E. elatior *(200 mg/kg b.w) (p < 0.05) (Figure 2).

### Lipid hydroperoxides

**Figure 3:**
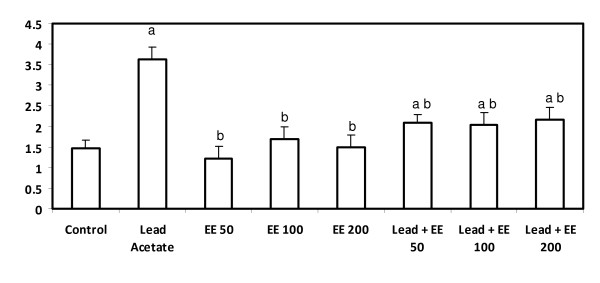
**Lipid hydroperoxide levels (nmol/mg protein) in control and *E. elatior *alone or in combination with lead acetate-treated rats**. Results are expressed as means ± S.D. of eight rats per group; EE - *Etlingera elatior *a - Significantly different from control - p < 0.05 b - Significantly different from lead alone treatment group - p < 0.05

The current results revealed that animals treated with lead acetate alone showed a significant increase in LPO compared to the other experimental groups (p < 0.05). Animals treated with *E. elatior *at the tested doses were comparable to the control regarding LPO level. The combined treatment of lead acetate plus the extract resulted in a significant decrease (p < 0.05) in LPO resulted from Lead exposure although these levels were still higher than the control group (Figure 3).

### Protein carbonyl content

**Figure 4:**
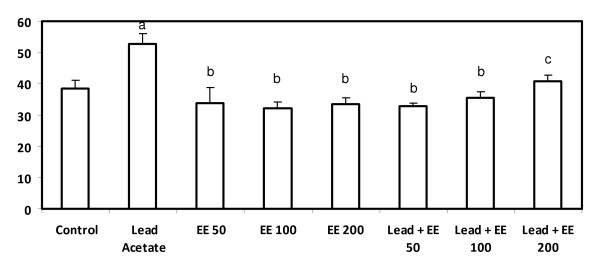
**Serum protein carbonyl content (nmol/mg protein) in control and *E. elatior *alone or in combination with lead acetate-treated rats**. Results are expressed as means ± S.D. of eight rats per group; EE - *Etlingera elatior *a - Significantly different from control - p < 0.05 b - Significantly different from lead alone treatment group - p < 0.05 c - Significantly different from lead + EE group - p < 0.05

A significant increase in serum protein carbonyl contents were recorded in lead-treated animals (p < 0.05). More than 30% increase in protein carbonyl content was seen in this group. The level of PCC decreased significantly when *E. elatior *was administrated to the lead acetate-treated groups (p < 0.05). Moreover, the decrease in PCC was pronounced in the group treated with *E. elatior *at the highest dose (200 mg/kg b.w). Though the level of PCC was significantly reduced with concurrent *E. elatior *treated rats, it did not significantly decrease below the control levels (Figure 4). It is of interest to mention that all groups treated with *E. elatior *alone or in combination with lead acetate did not show any significant differences among them or between the control groups.

### Superoxide dismutase

**Figure 5:**
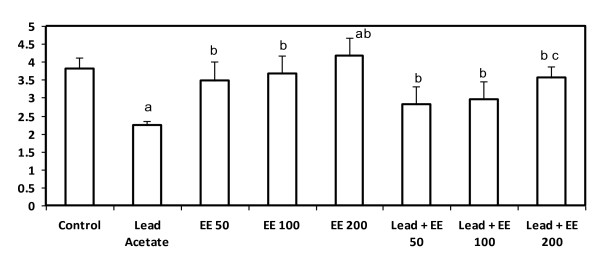
**Serum superoxide dismutase level (U/mg protein) in control and *E. elatior *alone or in combination with lead acetate-treated rats**. Results are expressed as means ± S.D. of eight rats per group; EE - *Etlingera elatior *a - Significantly different from control - p < 0.05 b - Significantly different from lead alone treatment group - p < 0.05 c - Significantly different from lead + EE group - p < 0.05

Serum superoxide dismutase showed a significant decrease (p < 0.05) in lead-treated group and a significant increase in *E. elatior *- treated groups compared to the control group (p < 0.05). Furthermore, the increase in SOD in *E. elatior *- treated groups was dose-dependent and it was higher than the control when the extract was administrated at the highest dose. On the other hand, the combined treatment with the extract and lead resulted in a significant improvement (p < 0.05) in SOD towards the control level although these treatments did not normalize SOD level except the group received the extract at the highest dose which was comparable to the control group (Figure 5).

### Glutathione peroxidase

**Figure 6:**
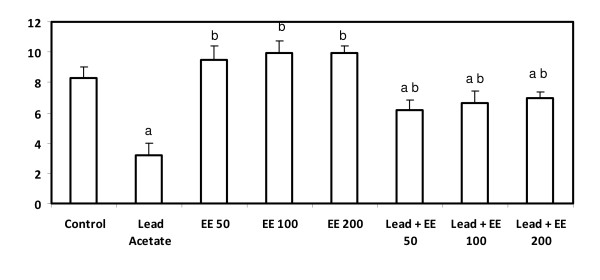
**Serum glutathione peroxidase level (nmol/mg protein) in control and *E. elatior *alone or in combination with lead acetate-treated rats**. Results are expressed as means ± S.D. of eight rats per group; EE - *Etlingera elatior *a - Significantly different from control - p < 0.05 b - Significantly different from lead alone treatment group - p < 0.05

The current study revealed that GPx was significantly decreased in animals treated with lead acetate alone (p < 0.05). Animals received the extract at the three tested doses showed a significant increase compared to the control group. Whereas, the combined treatment with lead and the extract at the three levels succeeded to induce a significant improvement (p < 0.05) in GPx, compared to lead alone-treated group, these treatments failed to normalize GPx (Figure 6).

### Glutathione S-transferase

**Figure 7:**
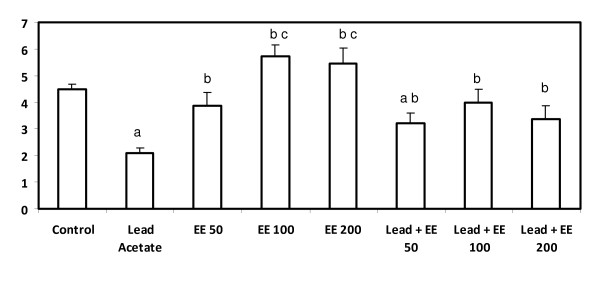
**Serum glutathione S-transferase level (*μ*mol/h/mg protein) in control and *E. elatior *alone or in combination with lead acetate-treated rats**. Results are expressed as means ± S.D. of eight rats per group; EE - *Etlingera elatior *a - Significantly different from control - p < 0.05 b - Significantly different from lead alone treatment group - p < 0.05 c - Significantly different between EE alone groups - p < 0.05

Animals treated with lead alone showed a significant decrease (p < 0.05) in GST level compared to the control group. The extract alone at lowest dose (50 mg/kg b.w) did not affect GST significantly whereas the other tested doses induced a significant increase compared to the control group. The combined treatment with lead and the extract resulted in a significant improvement (p < 0.05) in GST compared to lead acetate alone-treated group but failed to normalize it. The best result achieved in the group treated with lead and the extract at 100 mg/kg b.w. (Figure 7).

## Discussion and conclusion

Lead is known to cause oxidative damage in various tissues by bringing about imbalance in the generation and removal of reactive oxygen species [21-23]. Although the exact mechanisms by which lead induces oxidative stress in various tissues are not completely understood, evidence indicates that multiple mechanisms may be involved. Numerous plant products have been shown to have high potent antioxidant activity. Recently, bioflavonoids and polyphenols of plant origin have been used extensively for free radical scavenging and to inhibit lipid peroxidation [8,24].

The present study showed that lead acetate exposure in drinking water for 14 days resulted in severe oxidative stress. The selective dose of lead acetate used in the current study was based on previous literature [24,25]. There was increase in serum lipid hydroperoxide and protein carbonyl content and decrease in total antioxidants and antioxidant enzymes like superoxide dismutase, glutathione peroxidase and glutathione S-transferase levels. These observations confirm the findings of several studies, which reported alterations in antioxidant enzyme activities in lead exposed animals [6,9,26-28] and workers [29,30] and suggest a possible involvement of oxidative stress in the pathophysiology of lead toxicity. But it is not clear whether the changes in serum antioxidant enzymes are the cause of oxidative damage or a consequence of it.

There was a significant increase in serum lipid hydroperoxides and protein carbonyl contents after lead acetate exposure. Lead is known to produce oxidative damage in various organs by increasing lipid peroxidation [31,32]. Lipid peroxidation will inactivate cell constituents by oxidation and ultimately lead to loss of membrane integrity [6,33]. The observed increased lipid hydroperoxides in the current study in lead-treated group may be due to the formation of free radicals or through exhaustion of antioxidants, leading to oxidative stress. Intense lipid peroxidation caused by lead exposure may affect the mitochondrial and cytoplasmic membrane causing more severe oxidative damage in the tissues and consequently releasing lipid hydroperoxides into circulation [34,35]. Protein modifications elicited by direct oxidative attack lead to the formation of protein carbonyl derivatives and protein carbonyl content (PCC) is the most commonly used biomarker for protein oxidation [36-38]. The observed increase in protein carbonyl content in lead exposed rats confirms the oxidative stress induced by lead acetate in different tissues.

Treatment with lead acetate significantly decreased the activities of superoxide dismutase, glutathione peroxidase, glutathione S- transferase and total antioxidants level. These results are in agreement with previous reports [24,25,39]. Lead acetate is known to cause free radical damage in tissues by two mechanisms: Increased generation of ROS, including hydroperoxides, singlet oxygen and hydrogen peroxides, and by causing direct depletion of antioxidant reserves [22,40]. Superoxide dismutase, glutathione peroxidase and glutathione S-transferase enzymes take part in maintaining glutathione homeostasis in the tissues. These antioxidant enzymes are involved in the defense system against free radical mediated tissue or cellular damage after lead exposure [24,41,42]. The observed decrease in circulating antioxidants and decrease in serum total antioxidants confirm the lead acetate - induced depletion of antioxidants depletion.

In the present study, administration of ethanol extract of *Etlingera elatior *alone significantly increased the serum antioxidant enzymes. Treatment of *E. elatior *extract along with lead acetate treatment decreased the lead induced changes in lipid hydroperoxides and antioxidant enzyme levels. Results on blood lead levels (BLL) showed that lead acetate alone showed a significantly higher BLL compared to concurrent *E. elatior *and lead groups. Following oral intake, absorbed lead acetate is carried via blood to various tissues and more than 90% of blood lead is transported in the erythrocytes as lead phosphate [43,44]. This might have elevated blood lead levels in lead acetate ingested rats. The observed decrease in blood lead levels in *E. elatior *with lead acetate treatment group implies the possible chelating effect of *E*. *elatior *extract. However, this property of *Etlingera elatior *extract requires further study.

Relatively few data are available regarding the antioxidant effect of *E. elatior *plant extract. Although the flower and flower shoots of this plant have been used for food, there are no studies on the possible antioxidant effect of *E. elatior *flower extract till recently. Previous studies conducted in our laboratory showed that the inflorescence extract of the plant has powerful antioxidant activities against lead induced oxidative stress in liver and bone marrow [17,18]. *E. elatior *has decreased the lipid hydroperoxides and protein carbonyl contents after lead acetate exposure in the bone marrow and liver of rats. The study also showed that there was a significant increased production of antioxidant enzymes like super oxide dismutase, glutathione peroxidase and glutathione S - transferase with *E. elatior *treatment [17,18]. Most of the past studies on the antioxidant activities of *E. elatior *were confined to rhizomes [16,45] and leaves [11]. The rhizomes extract have been reported to have high antioxidant properties comparable to α- tocopherol. Habsah et al [16] reported that crude dichloromethane and methanol extracts of *E. elatior *leaves possessed antioxidant and antitumor promoting activity. In a comparative study Chan et al., [11] compared five species of *Etlingera *for their anti-oxidative properties. Among them *E. elatior *ranked highest in total phenolic content (TPC) and ascorbic acid equivalent antioxidant capability (AEAC) [46]. In a study on the antioxidant activities of inflorescences and rhizomes from *E. elatior*, inflorescence showed higher total phenolic content and antioxidant activities than rhizomes [11]. The salient finding from the current study regarding antioxidant effects is that *E. elatior *nearly normalized the perturbations of serum antioxidant enzymes, lipid hydroperoxides and protein carbonyl contents. The present study also showed a significant dose dependent effect of *E. elatior *against lead acetate - induced oxidative stress; the higher concentration of the extract (200 mg/kg) had produced a marked improvement in the total antioxidants and superoxide dismutase and produced a significant reduction in protein carbonyl contents in the lead treated group after 14 days confirming earlier reports [18].

The observed antioxidant activities of *E. elatior *flower extract against lead induced toxicity could be attributed to the antioxidant compounds present in the extract [17]. Phytochemical screening revealed significant amounts of polyphenolic and flavonoid compounds in the extract. These antioxidant compounds could have played a major role in scavenging the reactive oxygen species induced by lead acetate in the serum.

This study has shown that *E. elatior *ethanol extract from the inflorescence had significant antioxidant activity and caused a significant reversal of the lead - induced changes in the oxidative biomarkers in serum. Observed changes could be due to the different polyphenols, flavonoids, and flavones present in the extract. The results prove for the first time that *E. elatior *inflorescence extract has free radical scavenging and antioxidant properties.

## Competing interests

The authors declare that they have no competing interests.

## Authors' contributions

TJ carried out the laboratory studies, collection of plant materials, helped in analysis of data and preparation of manuscript, NH helped in animal experiments, laboratory studies and preparation of manuscript and analysis and interpretation of the data, SC involved in laboratory work, revising the manuscript and interpretation of data. All authors read and approved the manuscript.
